# Common mental health disorders and cognitive decline in a longitudinal Down syndrome cohort

**DOI:** 10.1192/bjo.2023.590

**Published:** 2023-11-03

**Authors:** Mina Idris, Fedal Saini, Sarah E. Pape, R. Asaad Baksh, Marie-Stephanie Cahart, André Strydom

**Affiliations:** Department of Forensic and Neurodevelopmental Sciences, Institute of Psychiatry, Psychology & Neuroscience, King's College London, London, UK; Centre for Neuroimaging Sciences, Institute of Psychiatry, Psychology & Neuroscience, King's College London, London, UK

**Keywords:** Down syndrome, trisomy 21, intellectual disability, Alzheimer's disease, common mental disorders

## Abstract

**Background:**

Down syndrome is the most common genetic cause of intellectual disability and Alzheimer's disease. In the general population, common mental disorders (CMDs), including anxiety, depression and obsessive–compulsive disorder, are linked to cognitive decline and higher risk for dementia. It is not known how CMDs affect longer-term cognitive outcomes in Down syndrome, and there is often diagnostic uncertainty in older people with Down syndrome and psychiatric comorbidity.

**Aims:**

To study the influence of CMDs on cognitive ability and whether they are related longitudinally to development of clinical signs of Alzheimer's disease in Down syndrome.

**Method:**

We followed 115 individuals with Down syndrome, 27 of whom were diagnosed with a CMD, over approximately 3 years. Changes in cognitive and behavioural outcomes between baseline and follow-up assessment were analysed, with comparisons made between those with and without a comorbid CMD. Age, gender, apolipoprotein E status and level of intellectual disability were included as covariates.

**Results:**

No significant association between presence of a CMD and poorer performance on cognitive tasks or informant-rated decline over time was observed (*P* > 0.05).

**Conclusions:**

Our results suggest that a diagnosis of a CMD does not have a significant negative effect on long-term cognitive or behavioural outcomes in individuals with Down syndrome. In individuals with stable or treated CMD, subsequent cognitive decline is likely indicative of Alzheimer's disease rather than a consequence of mental disorder.

Down syndrome is a neurodevelopmental disorder caused by triplication of chromosome 21 and is associated with an ultra-high risk of Alzheimer's disease with ageing.^1^ Common mental disorders (CMDs), defined in this study as a diagnosis of depression, anxiety or obsessive–compulsive disorder (OCD) including seasonal affective disorder (SAD), post-traumatic stress disorder (PTSD) and disruptive behaviour disorders, are relatively common in individuals with Down syndrome.^2^ In England, the prevalence of psychiatric diagnoses among the general population is estimated at 17%,^3^ whereas results from studies investigating the prevalence of CMDs among individuals with Down syndrome present a mixed picture. Studies with smaller samples have reported a prevalence ranging from 10.8 to 23.7%, depending on the classification used.^4^ Depression is one of the most frequently described mental health problems in individuals with Down syndrome, with prevalence rates ranging from 5.2 to 18.4%,^5^ compared with 3.3% in the general population.^3^ However, a recent population-based study indicated that mood and anxiety disorders are less common in individuals with Down syndrome than in the general population.^6^ Lastly, although younger individuals with Down syndrome are more prone to anxiety and externalising disorders, older individuals with Down syndrome are more likely to receive a diagnosis of depression.^7^

CMDs are associated with Alzheimer's disease-like cognitive decline in the general population.^8^ Anxiety is associated with poorer performance on memory tasks and has been linked to cognitive decline in older adults.^9^ Depression severity is associated with decline in episodic memory, language, working memory, executive function, as well as processing speed domains,^10^ which tends to persist even after remission of symptoms.^11^ Evidence has shown that cognitive impairment in older adults with depression is a strong predictor of dementia^12^ and that depression is associated with a twofold increase in the risk of developing dementia.^13^ Moreover, depressive and other psychiatric symptoms are also common among people with dementia and may be a response to early cognitive decline.^14^ The impact of CMDs on cognition has not been well-established in Down syndrome, despite depression and anxiety being common psychiatric conditions in Down syndrome.^15^ Some authors suggest that these psychiatric features might be regarded as prodromal symptoms of Alzheimer's disease.^16^ In addition, the presence of CMD symptoms may complicate diagnosis of Alzheimer's disease in people with Down syndrome, a population with lifelong cognitive impairments in whom it is difficult to establish the onset of cognitive decline. Clinicians are therefore often reluctant to diagnose dementia due to Alzheimer's disease in the presence of CMDs, and diagnosis of Alzheimer's disease may be significantly delayed in people with such comorbidity.^17^ It is therefore critical to understand the potential impact of CMDs on longer-term cognitive outcomes in people with Down syndrome, as this could improve diagnostic certainty and allow for earlier access to treatment and better support.

## Aims

We aimed to understand whether the presence of CMDs was associated with a decline in measures of cognition and adaptive behaviours in people with Down syndrome longitudinally, compared with those without a diagnosis of CMD.

## Method

### Study design

The London Down Syndrome (LonDownS) Consortium study is a longitudinal study of people with Down syndrome. All participants undergo extensive neuropsychological examination using the LonDownS Consortium battery, which has been described in previous studies.^18^ The battery involves a series of neuropsychological tests measuring memory, language, executive function and motor skills. Semi-structured interviews and questionnaires measuring adaptive behaviour and Alzheimer's disease-related decline are also administered to each participant's caregiver, alongside questions about demographic details and medical history. Participants and carers completed the LonDownS assessment at baseline (*T*_1_) and subsequent follow-up (*T*_2_) an average of 36 months after the baseline assessment.

The authors assert that all procedures contributing to this work comply with the ethical standards of the relevant national and institutional committees on human experimentation and with the Helsinki Declaration of 1975, as revised in 2008. All procedures involving human subjects/patients were approved by the North-West Wales Research Ethics Committee (13/WA/0194).

### Inclusion criteria

Inclusion criterion for the initial LonDownS Consortium study was all adults with Down syndrome over the age of 16. Capacity was evaluated for participants at each assessment and written informed consent was obtained from all participants where possible. For those that lacked capacity, their carers acted as consultees. Carers were required to indicate their decision about the participant's inclusion based on their knowledge of the participant and their wishes, in accordance with the UK Mental Capacity Act 2005. Down syndrome diagnosis was genetically confirmed using saliva or blood samples.

### Classification of CMD and exclusion criteria

At baseline, a comprehensive medical history was obtained in collaboration with caregivers. Participants with an ongoing diagnosis of depression, anxiety, OCD and related conditions such as SAD, PTSD and behavioural disorders at the time of their cognitive assessment were categorised as having a diagnosis of a CMD. In addition, participants with CMD-like symptoms reported by their doctor, and participants who were also prescribed medication typically used to treat CMDs, including selective serotonin reuptake inhibitors (SSRIs) and serotonin and noradrenaline reuptake inhibitors (SNRIs) such as citalopram, fluoxetine, mirtazapine, sertraline and trazodone, were included. These participants were classed as having a CMD. Those without CMDs were therefore defined as individuals with no diagnosis, symptoms or prescription of relevant medications at the baseline visit. To focus on longer-term cognitive impact rather than acute effects of CMDs, participants who did not have a CMD at baseline but who had developed CMD by the time of their follow-up visit were removed from analyses. Finally, to be eligible for inclusion in this analysis, participants were required to have no diagnosis of dementia at baseline.

### Cognitive and behavioural outcome measures

Cognitive task outcomes were measures of memory (Cambridge Neuropsychological Test Automated Battery Paired Associates Learning (CANTAB PAL) and Cambridge Cognitive Examination (CAMCOG) Orientation subscale), general verbal and non-verbal cognitive abilities (Kaufman Brief Intelligence Test – Second Edition (KBIT-2) and CAMCOG Orientation subscale), executive functioning (CAMCOG Verbal Fluency subscale and Tower of London test) and attention (CANTAB Simple Reaction Time (CANTAB SRT)). These tasks were chosen to assess skills associated with brain areas most affected in both CMDs and Alzheimer's disease; they are suited to a range of ages and abilities, including non-verbal individuals and have good test–retest reliability^18^ (Table 1).

**Table 1:** Cognitive outcome measures

	Test	Description	Outcome measures used in this analysis
Memory functions assessments	Cambridge Neuropsychological Test Automated Battery Paired Associates Learning (CANTAB PAL)^20^	Participants observe and recall pattern locations to test visuospatial short-term memory.	First trial memory score: 0–26, representing the number of times a participant chose the correct box on their first attempt when recalling the pattern locations, calculated across all assessed trials. A higher score represents better performance.
	Cambridge Cognitive Examination (CAMCOG) Orientation subscale^21^	Participants are asked their full name, the day of the week, the month, the year, where they are and the nearest city/town. Clues are given (for a lower score) if there is incorrect/no response.	Orientation total score: 0–12, with a higher score meaning better performance.
General cognitive ability assessments	Kaufman Brief Intelligence Test – Second Edition (KBIT-2)^22^	Participants are required to identify correct answers in two verbal subtests (verbal knowledge and riddles) plus one non-verbal subtest (matrices).	Total IQ score, lowest score 40, highest score 160.
Executive function assessments	Cambridge Cognitive Examination (CAMCOG) Verbal Fluency subscale^21^	Participants are asked to name as many animals as they can in 1 min to test frontal function.	Raw score number named (novel): 0-NA. Those naming a higher number of animals, not including intrusions and repetitions, sored the best.
	Tower of London^23^ adapted version^24^	Participants move beads on a board to match presented configurations to test working memory and planning.	Total score: 0–10, with a higher score meaning better performance.
	Cambridge Neuropsychological Test Automated Battery Simple Reaction Time (CANTAB SRT)^20^	Reaction time test requiring participants to press a button in response to a white square appearing.	Mean latency and standard deviation of latency of reaction time (ms) in 100 trials. Participants with smaller mean and standard deviation of reaction time performed best.

The Short Adaptive Behavior Scale (SABS)^19^ is an informant questionnaire that was used to assess changes in behaviours between *T*_1_ and *T*_2_. The SABS measures adaptive behaviours across three domains: personal self-sufficiency, community self-sufficiency and personal-social responsibility. The SABS results at baseline assessment were compared with the follow-up assessment results, with lower scores at follow-up suggesting functional decline.

### Statistical analysis

Change scores were calculated for cognitive tasks and questionnaire scores over the follow-up period for each participant. The score at *T*_1_ was subtracted from the score at *T*_2_. Multiple linear regressions were performed on change in cognitive task and SABS scores over time to determine whether individuals diagnosed with a CMD at baseline showed negative change over time compared with individuals without a CMD diagnosis.

In the cognitive task analysis, the outcome measure was change in cognitive task scores on each of the cognitive tasks between visits. For the analysis of adaptive behavioural changes, the outcome measure was change in SABS score in three summary domains – personal self-sufficiency, community self-sufficiency and personal-social responsibility – as well as the total score. In all the statistical models the main predictor was CMD.

Relevant additional predictors were included in each model: these were age, gender and level of intellectual disability. Apolipoprotein E (APOE) status was also included, as people with Down syndrome who are APOE ɛ4 allele carriers show earlier neurodegenerative changes and clinical symptoms of Alzheimer's disease.^25^ All statistical analyses were then performed using R (version 4.0.5 for MacOS)^26^ in RStudio 1.4.1106 with a significance level of 0.05.

## Results

### Participants

**Fig. 1:**
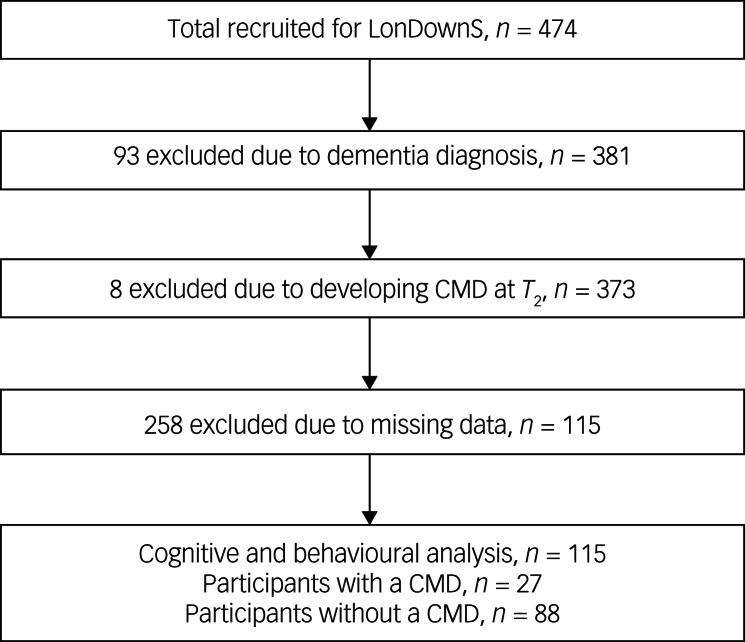
Flow chart showing participant exclusion and final cognitive data sample size. LonDownS, London Down Syndrome Consortium study. CMD, common mental disorder; *T*_2_, follow-up assessment.

From the total number of individuals recruited for LonDownS (*n* = 474), 94 were excluded from the analyses as they presented with a dementia diagnosis at the initial assessment and a further 7 were excluded as they had developed a CMD by follow-up. These participants were excluded in order to focus specifically on the effects of mental health conditions over time to prevent the potential confounding factor of diminished task scores caused by dementia-related illness or subsequent development of CMDs. Of the remaining 373 participants, 115 had complete cognitive task and behavioural data at baseline and after at least 2 years, 27 (23.48%) of whom were classed as having a CMD (Fig. 1; see Table 2 for the demographic characteristics of the final sample).

**Table 2:** Participant demographics by common mental disorder (CMD) status at baseline (*n* = 115)

Demographic	All participants	Participants with a CMD	Participants without a CMD
Total, *n* (%)	115 (100.00)	27 (23.48)	88 (76.52)
Gender, *n* (%)			
Female	54 (46.96)	13 (11.30)	41 (35.65)
Male	61 (53.04)	14 (12.17)	47 (40.87)
Ethnicity, *n* (%)			
White	101 (87.83)	25 (21.74)	76 (66.09)
Black	7 (6.09)	1 (0.87)	6 (5.22)
Indian	2 (1.74)	0 (0.00)	2 (1.74)
Pakistani	1 (0.87)	0 (0.00)	1 (0.87)
Mixed	2 (1.74)	0 (0.00)	2 (1.74)
Other/NA	2 (1.74)	1 (0.87)	1 (0.87)
Age at initial assessment, years: mean (s.d.) (range)	38.03 (12.61) (16–66)	43.30 (10.09) (18–66)	36.42 (12.92) (16–63)
Length of follow-up, months: mean (s.d.) (range)	36.64 (18.58) (20–100)	29.85 (15.34) (23–97)	38.73 (19.06) (20–100)
Level of intellectual disability, *n* (%)			
Mild	47 (40.87)	15 (13.04)	32 (27.83)
Moderate	59 (51.30)	12 (10.43)	47 (40.87)
Severe	9 (7.83)	0 (0.00)	9 (7.83)
APOE status, *n* (%)			
ε4 carrier	27 (23.48)	3 (2.61)	24 (20.87)
Non-ε4 carrier	81 (70.43)	22 (19.13)	59 (51.30)
Missing	7 (6.09)	2 (1.74)	5 (4.35)

APOE, apolipoprotein E.

### Changes in cognitive and behavioural scores over time

Both groups (i.e. with a CMD diagnosis and without a CMD diagnosis) showed differences in mean change scores over time (Table 3). Both groups showed declining scores for the Tower of London, CANTAB SRT mean latency, CAMCOG Verbal Fluency and CAMCOG Orientation between the baseline and follow-up visits. Participants with a CMD had greater decline in Verbal Fluency score than those without. However, both groups also showed improved performance in KBIT IQ, CANTAB PAL and CANTAB SRT standard deviation scores between visits. To examine associations between CMD and cognitive score, separate multiple regression analyses were used to model whether having a diagnosed CMD significantly predicted participants’ change scores on each cognitive task, controlling for age, gender, level of intellectual disability and APOE status (Table 4). Results showed that presence of a CMD as a predictor of change in cognitive task score was not statistically significant for any tasks (*P* > 0.05). Thus, no association was found between presence of a CMD and task change score.

**Table 3:** Mean task scores in affected versus unaffected individuals and change over time

	Participants with a common mental disorder			Participants without a common mental disorder		
Task	*T*_1_	*T*_2_	Change^a^	*T*_1_	*T*_2_	Change^a^
Tower of London	7.21	7.04	**−0.16**	6.93	6.47	**−0.45**
CAMCOG Verbal Fluency	11.15	10.85	**−0.30**	10.06	9.98	**−0.08**
CAMCOG Orientation	9.56	9.22	**−0.33**	9.46	8.73	**−0.74**
KBIT IQ	49.31	50.52	**1.21**	46.99	48.13	**1.14**
CANTAB PAL First Trial Memory	9.82	11.06	**1.24**	8.74	9.12	**0.38**
CANTAB SRT mean latency	660.19	675.86	**15.67**	757.01	853.05	**96.04**
CANTAB SRT standard deviation	322.29	299.49	**−22.80**	372.66	368.09	**−4.57**

*T*_1_, baseline; *T*_2_, follow-up; CAMCOG, Cambridge Cognitive Examination; KBIT, Kaufman Brief Intelligence Test – Second Edition; CANTAB, Cambridge Neuropsychological Test Automated Battery; PAL, Paired Associates Learning; SRT, Simple Reaction Time.

a. Bold denotes significance at *P* ≤ 0.05.

**Table 4:** Cognitive tasks score multiple regression^a^

Task	*B*	95% CI	*P*
Tower of London	0.11	−0.70 to 2.07	0.33
CAMCOG Verbal Fluency	−0.01	−1.52 to 1.39	0.93
CAMCOG Orientation	0.08	−0.61 to 1.44	0.43
KBIT IQ	0.03	−2.86 to 3.68	0.80
CANTAB PAL First Trial Memory	−0.18	−3.70 to 0.42	0.12
CANTAB SRT mean latency	−0.08	−193.72 to 100.10	0.53
CANTAB SRT standard deviation	0.03	−64.89 to 80.85	0.83

CAMCOG, Cambridge Cognitive Examination; KBIT, Kaufman Brief Intelligence Test – Second Edition; CANTAB, Cambridge Neuropsychological Test Automated Battery; PAL, Paired Associates Learning; SRT, Simple Reaction Time.

a. All models were adjusted for age, gender, level of intellectual disability and apolipoprotein E status.

Both groups showed differences in behavioural change scores over time (Table 5). Both groups showed improvement in mean behavioural scores between visits, and those with a CMD showed greater improvement (+16.73) than those without (+9.84) in the SABS total. Multiple linear regression was used to analyse associations between CMDs and scores representing behavioural changes reported on the SABS over time (Table 6). No significant effect of CMD state at baseline on SABS change score over time was demonstrated (*P* > 0.05).

**Table 5:** Mean Short Adaptive Behavior Scale (SABS) scores in affected and unaffected individuals and change over time

Measure	Participants with a common mental disorder			Participants without a common mental disorder		
	*T*_1_	*T*_2_	Change	*T*_1_	*T*_2_	Change
SABS Domain A	24.07	28.59	4.52	26.06	29.28	3.23
SABS Domain B	21.38	30.22	8.84	23.68	27.40	3.72
SABS Domain C	20.37	23.35	2.98	19.60	22.38	2.78
SABS total	65.58	82.31	16.73	69.34	79.18	9.84

**Table 6:** Short Adaptive Behavior Scale (SABS) score multiple regression^a^

Measure	*B*	95% CI	*P*
SABS Domain A	−0.03	−4.87 to 3.67	0.78
SABS Domain B	0.02	−6.22 to 7.47	0.86
SABS Domain C	−0.10	7.39 to 2.37	0.31
SABS total	−0.04	−17.70 to 12.24	0.72

a. All models were adjusted for age, gender, level of intellectual disability and apolipoprotein E status.

In summary, presence of a CMD was not significantly associated with cognitive and behavioural changes over time and there were no significant differences in change scores between participants with and without a CMD.

Seven participants were diagnosed with a CMD between their baseline and follow-up assessments. Among those who completed tasks on both visits, most participants with new onset of a CMD performed worse overall (Table 7). However, there are incomplete data for this subset of participants because those with unstable illness were unable to complete all tasks, and therefore these participants were not included in the above regressions.

**Table 7:** Change scores of participants who received a diagnosis of common mental disorder between visits

Age, years	Tower of London	CAMCOG Verbal Fluency	CAMCOG Orientation	KBIT IQ	CANTAB PAL First Trial Memory	CANTAB SRT mean latency	CANTAB SRT standard deviation	SABS total
49		2	**−5**	0	**−5**			52
44	0	5	0	**−6**	0	−167.19	−137.45	10
58								**−6**
41								
44				**−2**				
21	**−1**	6	**−1**	**−2**	**−7**	**190**.**97**	**98**.**67**	
29	2	**−8**	**−2**	**−12**	**−2**			**−7**

CAMCOG, Cambridge Cognitive Examination; KBIT, Kaufman Brief Intelligence Test – Second Edition; CANTAB, Cambridge Neuropsychological Test Automated Battery; PAL, Paired Associates Learning; SRT, Simple Reaction Time; SABS, Short Adaptive Behavior Scale.

a. Bold denotes declining scores.

## Discussion

### Main findings

This longitudinal study investigated associations between CMDs and cognitive decline in people with Down syndrome without dementia. We hypothesised that the presence of CMDs may be a significant risk factor for increased Alzheimer's disease-related cognitive decline over time in this population. We found no significant association between the presence of a stable or treated CMD at baseline and cognitive or functional decline over time; however, those with new onset of a CMD during the follow-up tended to have worse scores on cognitive and/or functional ability assessments.

### Interpretation of our findings

CMDs, particularly depression, are known to be associated with cognitive impairments, which can significantly affect a person's independent functioning and quality of life in the short term. CMDs are also considered risk factors for Alzheimer's disease, with depression earlier in life being associated with risk of dementia in the general population.^9^ Depression and anxiety symptoms are also frequently observed alongside mild cognitive impairment in the early stages of Alzheimer's disease and have been considered as prodromal features of Alzheimer's disease.^14^ Previous studies in people with Down syndrome have found associations between depression and impairment in adaptive behaviour,^27–29^ with the severity of impairment being more pronounced in those with early-onset depression.^27^ Notably, a year of pharmacological treatment has demonstrated efficacy in mitigating these disturbances, even without significant changes in depressive symptomatology.^29^ Furthermore, depression in individuals with Down syndrome is associated with impairments in daily living activities, social abilities, lower mental age and poorer memory performance.^28,30^ Finally, speech impairment has been found to negatively correlate with depression and anxiety.^31^

In our study, the presence of stable and/or treated CMDs in individuals with Down syndrome was not significantly associated with cognitive decline or behavioural changes over a 3-year period. A key factor in our study is the use of clinically confirmed diagnoses of CMDs by the participants’ doctor, meaning that these mental health conditions have been diagnosed and that these individuals have likely been offered treatment, either with medication or with psychological or psychosocial interventions. It has been previously shown in Down syndrome that 1 year of antidepressant pharmacotherapy produced a significant recovery in adaptive functioning even though depressive symptoms were still present.^29^ Half of our participants were receiving pharmacological treatment, and this may have mitigated the cognitive decline that can be associated with CMDs in these individuals. In the general population, antidepressants have been shown to help reduce cognitive deficits associated with CMDs. For instance, a systematic review and meta-analysis showed that SSRIs have beneficial effects on attention, executive function, memory and processing speed in people with depression.^32^ A recent clinical trial showed that vortioxetine, a multimodal antidepressant, was associated with significant improvement of executive function, attention, learning, memory and processing speed in adults with depression from the general population.^33^ SNRIs can also be effective in improving cognitive functions such as declarative and working memory,^34^ verbal learning^35^ and psychomotor speed.^36^ Pharmacological interventions may therefore have mitigated the detrimental cognitive effect potentially associated with CMDs in our sample.

Another factor that may have influenced the results of the study is related to the developmental trajectory of cognition in Down syndrome. The cognitive profile in Down syndrome is typically characterised by growth persisting throughout adolescence and early adulthood, followed by a gradual loss of ability,^37^ which is frequently associated with neurodegenerative processes.^31^ Therefore, the potential negative influence of CMDs on cognition, if present, may be less pronounced in individuals with Down syndrome.

Our study confirms that CMDs are common in people with Down syndrome. In our cohort, CMDs that occurred in participants without dementia were not related to cognitive and functional decline over time. However, the seven participants that developed a CMD between baseline and follow-up appeared to show some cognitive decline. Whether this was due to acute cognitive effects of CMDs or whether these individuals presented instead with neuropsychiatric prodromal symptoms of dementia is not clear. Of these seven participants, five were aged 41 and older, and therefore may have been experiencing some prodromal dementia symptoms. Nonetheless, our data suggest that CMDs may not be a significant additional risk factor for cognitive decline in adults with Down syndrome in the long term, particularly if well managed, in the context of an overwhelming genetic risk for Alzheimer's disease due to amyloid precursor protein (*APP*) gene triplication, although those with acute presentations may benefit from optimisation of treatment and cognitive surveillance.

### Strengths and limitations

Our study had several strengths. By using each participant's own baseline, we were able to control for the effects of baseline cognitive and functional skills and track changes for that individual, thus improving the sensitivity of the assessment. We used neuropsychological test scores and informant ratings to consider changes across a wide range of domains. In addition, we included participants with severe intellectual disability, who are often excluded from research. The follow-up was longer than in most other studies of cognitive decline in Down syndrome, and although it is possible that decline in those with CMDs might have been demonstrated over a longer time period, this is unlikely, as in naturalistic studies of decline in people with Down syndrome aged 35 and older, decline has been observed within 2 years.^38^ This is also in keeping with previous studies testing the hypothesis of depression and anxiety as prodromal features of Alzheimer's disease in the general population that considered a similar time window between the diagnosis of CMDs and the onset of Alzheimer's disease (e.g.^39,40^).

However, this study also has some limitations. Although this is a longitudinal study with a relatively large sample, those included at baseline may have been under-representative of those with more severe episodes of mental illness. Another limitation of this study is that the use of SSRI and SNRI prescription as an indicator of CMD in individuals with Down syndrome may not always accurately reflect the presence of a CMD, as these medications are occasionally prescribed for other types of mental health condition, such as chronic pain disorder, eating disorders and severe mental illnesses. Also, we did not complete our own clinical assessments of the presence of CMD, but rather relied on diagnoses obtained from medical histories. Furthermore, we could not examine potential age differences in the impact of CMDs on cognition, as our sample was not large enough to allow for age-related grouping. It should be noted that our study did not specifically account for the presence of autism spectrum disorder (ASD), which can coexist and show overlapping symptoms with OCD in Down syndrome. ASD traits can be a confounding factor when examining the potential influence of CMDs on cognition, and future studies should take this into account. Finally, there were modest inter-participant differences in follow-up period that we did not statistically control for; however, we have controlled for participants’ age, as this has been shown to be one of the most important predictors of decline in analyses of Down syndrome studies of Alzheimer's disease.

### Considerations for clinical practice

Despite widespread awareness of the high risk of Alzheimer's disease in people with Down syndrome, there remain many factors affecting diagnostic certainty when assessing for Alzheimer's disease in clinical settings. Some of these challenges are related to difficulties with self-report of symptoms, diminished communication skills and differences in psychopathology presentation.^41^ Others are driven by the reliance on data from informants who may have limited historical knowledge of an individual.^42^ A significant consideration for clinicians is whether symptoms of cognitive decline in those with a history of mental health issues are related to a CMD rather than early symptoms of Alzheimer's disease. Our findings suggest that although symptom overlap between CMDs and Alzheimer's disease is acknowledged,^43^ CMDs are not associated with significant objective cognitive decline over the longer term. A diagnosis of Alzheimer's disease should therefore be considered in those presenting with typical symptoms even in the presence of a comorbid CMD. This may reduce delays in both assessment and provision of support. However, evidence of cognitive decline from an individual's baseline would be important for an accurate diagnosis of Alzheimer's disease, and acute presentations of CMDs need to be treated before Alzheimer's disease can be diagnosed.

In conclusion, we found that stable and treated CMDs were not associated with longitudinal Alzheimer's disease-like cognitive decline and functional changes in adults with Down syndrome. This highlights the importance of baseline cognitive assessments being routinely offered for all people with Down syndrome prior to Alzheimer's disease onset. Further research is needed to investigate CMDs in earlier life and their later impact on cognition and behaviour, behavioural and psychiatric presentations as prodromal symptoms of dementia and the impact of treatment of CMDs in the Down syndrome population on the onset of Alzheimer's disease symptoms. Finally, the association between diagnosis of dementia and presence of CMDs is an area that warrants further study.

## Data Availability

The data that support the findings of this study are available from the corresponding author M.I. on request. The data are not publicly available as they contain information that could compromise the privacy of research participants.
